# Preprocedural screening for multidrug-resistant organisms in endoscopic retrograde cholangiopancreatography: an international, multicentre, cross-sectional observational study

**DOI:** 10.1016/j.eclinm.2025.103627

**Published:** 2025-11-13

**Authors:** Koen van der Ploeg, Margreet C. Vos, Hardik Rughwani, Juliëtte A. Severin, Richard A.J. Post, D. Nageshwar Reddy, Sadhana Yelamanchili Veturi, Mitnala Sasikala, Sana Fathima Memon, Alessandro Repici, Marco Spadaccini, Matteo Colombo, Marta Andreozzi, Bryan A. Stevens, Rohit Das, Adam Slivka, Bibi C.G.C. Mason-Slingerland, Marco J. Bruno

**Affiliations:** a Department of Gastroenterology and Hepatology, Erasmus MC University Medical Center, Rotterdam, The Netherlands; b Department of Medical Microbiology and Infectious Diseases, Erasmus MC University Medical Center, Rotterdam, The Netherlands; c Department of Gastroenterology, AIG Hospitals, Hyderabad, India; d Department of Biostatistics, Erasmus MC University Medical Centre, Rotterdam, The Netherlands; e Department of Epidemiology, Erasmus MC University Medical Centre, Rotterdam, The Netherlands; f Department of Clinical Microbiology, AIG Hospitals, Hyderabad, India; g Department of Translational Research, AIG Hospitals, Hyderabad, India; h Department of Biomedical Sciences, Humanitas University, Milan, Italy; i Department of Endoscopy, Humanitas Clinical and Research Center IRCCS, Rozzano, Italy; j Department of Pathology, University of Pittsburgh, Pittsburgh, PA, USA; k Department of Gastroenterology, Hepatology, and Nutrition, University of Pittsburgh Medical Center, Pittsburgh, PA, USA

**Keywords:** Endoscopic retrograde cholangiopancreatography, Microbial drug resistance, Cross infection, Healthcare associated infections, Gastrointestinal endoscopes

## Abstract

**Background:**

Endoscopic retrograde cholangiopancreatography (ERCP) carries a risk of patient-to-patient transmission of multidrug-resistant organisms (MDROs) via contaminated duodenoscopes. Data on preprocedural MDRO carriage are limited and essential for guiding targeted prevention strategies, including the potential use of single-use duodenoscopes. This study assessed MDRO carriage among patients undergoing ERCP across four countries.

**Methods:**

In this international, multicentre, cross-sectional observational study, adults undergoing ERCP in tertiary care centres in the Netherlands, India, Italy, and the United States were screened for MDROs using preprocedural rectal and throat-nose swabs. Consecutive adult patients (aged ≥18 years) undergoing ERCP, regardless of indication, were eligible for inclusion. Exclusion criteria included cases in which ERCP was not performed, a duodenoscope was not used, or the rectal swab was not collected. MDROs screened included extended-spectrum beta-lactamase-producing *Enterobacterales* (ESBLE-E), carbapenemase-producing *Enterobacterales* (CPE), carbapenemase-producing *Pseudomonas aeruginosa* (CPPA), resistant *Acinetobacter calcoaceticus baumannii* complex (Acb-complex), vancomycin-resistant *Enterococcus faecium* (VRE), and methicillin-resistant *S. aureus* (MRSA). The primary outcome was the prevalence of MDRO among patients undergoing ERCP defined by growth of these organisms on preprocedural swab cultures. Secondary outcome was identification of risk factors for MDRO carriage through multivariable logistic regression analysis. Clinical and procedural variables, as well as microbiological results, were systematically retrieved from patients’ medical records and institutional laboratory databases. This study is registered at ClinicalTrials.gov, NCT05303662.

**Findings:**

Between Jan 22, 2022, and Oct 09, 2024, 1244 patients were enrolled, of whom 798 (64.1%) were male and 446 (35.9%) were female. Among all participants, 462 (37.1%, 95% CI 34.5–39.9) carried an MDRO. Prevalence was highest in India (290/349, 83.1%, 78.8–86.7) and lowest in the Netherlands (37/343, 10.8%, 7.9–14.5), with intermediate rates in Italy (66/209, 31.5%, 25.7–38.2) and the United States (69/343, 20.1%, 16.2–24.7). MDRO species and resistance mechanisms varied by country. ESBL-E were most prevalent in India (245/349, 70.2%, 65.2–74.8) compared with Italy (42/209, 20.1%, 15.2–26.0), the Netherlands (35/343, 10.2%, 7.4–13.9), and the United States (15/343, 4.4%, 2.7–7.1) (p < 0.001). CPE were detected in 82/349 (23.5%, 19.4–28.2) patients in India but were uncommon in Italy (8/209, 3.8%, 1.9–7.4), rare in the United States (3/343, 0.9%, 0.3–2.5), and nearly absent in the Netherlands (1/343, 0.3%, 0.0–1.6) (p < 0.001). CPPA was only detected in 1/349 (0.3%, 0–1.6) patient in India, with none in the other centres (p = 0.46). Resistant Acb-complex was only detected in 1/209 (0.5%, 0–2.7) patient in Italy (p = 0.18). VRE was most frequent in the United States (37/343, 10.8%, 7.9–14.5), followed by Italy (20/209, 9.6%, 6.3–14.3) and India (26/349, 7.4%, 5.1–10.7), and was not detected in the Netherlands (p < 0.001). MRSA was detected primarily in the United States (27/343, 7.9%, 5.5–11.2), with lower prevalence in Italy (6/209, 2.9%, 1.3–6.1), India (5/349, 1.4%, 0.6–3.3), and the Netherlands (1/343, 0.3%, 0.0–1.6) (p < 0.001). Significant risk factors for MDRO carriage included country of inclusion, with higher odds observed in India (aOR 99.14, 95% CI: 48.21–203.86, p < 0.001) and Italy (aOR 6.58, 95% CI: 3.57–12.14, p < 0.001) compared with the Netherlands (reference), while the association for the United States was not statistically significant (aOR 1.61, 95% CI: 0.82–3.14, p = 0.17). Other significant risk factors were chronic lung disease (aOR 1.75, 95% CI:1.07–2.86, p = 0.025), congestive heart failure (aOR 2.20, 95% CI: 1.33–3.64, p = 0.002), and prior use of penicillins (aOR 1.66, 95% CI: 1.05–2.63, p = 0.031).

**Interpretation:**

This study shows global heterogeneity in MDRO carriage among patients undergoing ERCP, reflecting underlying differences in antimicrobial resistance, healthcare infrastructure, and infection control practices. Strategies to prevent endoscope-associated transmission should therefore be tailored to local resistance patterns rather than adopting a universal approach. Preprocedural screening and targeted infection prevention strategies should be integrated within broader, region-specific infection control frameworks. The study's limitations should be considered when interpreting the findings, including restriction to tertiary care centres, incomplete enrolment data, and inter-site variability in patient characteristics and microbiological methods, which may affect generalisability. Nevertheless, the results provide a robust foundation for developing targeted, evidence-based interventions and for future research evaluating the clinical value, cost-effectiveness, and sustainability of preprocedural screening and infection prevention policies across diverse healthcare settings.

**Funding:**

Boston Scientific International and Copan Italia SpA.

**Research in context**

**Evidence before this study**

To assess the existing evidence, we searched PubMed for articles published in any language up to September 30, 2021, using combinations of the terms “Cholangiopancreatography, Endoscopic Retrograde”, “Duodenoscope”, “Drug Resistance, Multiple”, “Methicillin-resistant *Staphylococcus aureus*”, “Vancomycin Resistant *Enterococci*”, “Beta-Lactam Resistance”, “Carbapenem-Resistant Enterobacteriaceae”, “*Acinetobacter baumannii*”. Only three United States–based single-centre studies reported on a limited number of multidrug-resistant organism (MDRO) types identified through rectal screening in patients undergoing endoscopic retrograde cholangiopancreatography (ERCP). To our knowledge, no multicentre studies have systematically evaluated the prevalence and risk factors for MDRO carriage across diverse geographic regions in this population.

**Added value of this study**

In this large observational study of patients undergoing ERCP across four countries we illustrate that there is significant variation in prevalence, species, and resistance mechanisms of MDRO in this patient group. This data serves as critical input for evaluating the clinical utility, cost-effectiveness and environmental consequences of preventive strategies, including the use of single-use duodenoscopes.

**Implications of all the available evidence**

The high and variable prevalence of MDRO carriage underscores the need for targeted, region-specific infection preventive strategies. Preprocedural screening may enable tailored interventions to reduce the risk of duodenoscope-related transmission and outbreaks. Key study limitations include restriction to tertiary care centres, non-uniform site documentation of screening and exclusion counts, and inter-site variability in patient characteristics and microbiological methods. These factors may affect representativeness and generalisability.

## Introduction

Endoscopic retrograde cholangiopancreatography (ERCP) is the gold standard intervention for treating various biliary, liver, and pancreatic diseases. In the United States (US), approximately 650,000 ERCP procedures are performed annually.^1^ Over the past two decades, it has become evident that ERCP carries a risk of patient-to-patient transmission of pathogens via reusable duodenoscopes.^2^ These transmissions can result in duodenoscope-associated colonisation (DAC), which may progress to duodenoscope-associated infection (DAI).^3^ This risk is particularly concerning when multidrug-resistant organisms (MDROs) are involved.^2^ Outbreaks of DAIs have spurred extensive efforts by government agencies, healthcare organisations, and endoscope manufacturers to find solutions and preventive strategies for duodenoscope contamination.^4^^,^^5^

Reusable duodenoscopes require reprocessing after each use, following lengthy and complex protocols.^6^ Because of heat-sensitive components, steam sterilisation is not feasible. Alternative low-temperature methods, such as ethylene oxide sterilisation, significantly extend reprocessing time and pose safety risks due to carcinogenicity.^7^ Moreover, none of the current reprocessing protocols guarantees a zero-contamination rate.^8^ The Food and Drug Administration (FDA) has issued multiple safety communications urging a shift toward duodenoscopes with disposable components or fully single-use designs.^4^ While single-use duodenoscopes (SUD) eliminate the risk of cross-contamination, adoption is limited by cost and environmental concerns.^9^ Initial analyses regarding cost-effectiveness and environmental impact of SUD suggest that the price would need to decrease by up to tenfold.^9^^,^^10^ Additionally, using SUD would generate 24–47 times more carbon dioxide and 40%–400% more waste.^11^^,^^12^ However, full life cycle analyses are pending. Nevertheless, SUD may be justifiable for high-risk populations, such as patients colonised with an MDRO, where preventing transmission and difficult-to-treat infections may outweigh the disadvantages.^13^

The global prevalence of MDROs is increasing, with an estimated 4.71 million deaths linked to these pathogens in 2021.^14^ However, there are significant variations in MDRO prevalence across countries, age groups, patient populations and healthcare settings.^14^ Given that many patients undergoing ERCP have comorbidities and frequent healthcare exposure, the prevalence of MDROs in this population is likely higher than in the general population.^15^ The primary objective of this study was to assess MDRO carriage among patients undergoing ERCP in countries with differing expected prevalence rates. The secondary objective was to identify risk factors for preprocedural MDRO carriage in this population.

## Methods

### Study design

This multicentre observational study was conducted between Jan 22, 2022, and Oct 09, 2024 across four tertiary care centres in the Netherlands, India, Italy, and the US. The medical ethics committee of the study centre in the Netherlands (CPB/aj/MEC-2021-0768) and the institutional ethics committees of the study centres in India (AIG/IEC-BH&R16 (b)/07.2021-03), Italy (CE Humanitas ex D.M. 8/2/2013; 566/22), and the United States (STUDY22030042) each approved the study. The study was registered at ClinicalTrials.gov (ID: NCT05303662). The study was conducted and reported in accordance with the Strengthening the Reporting of Observational Studies in Epidemiology (STROBE) guidelines.

### Outcomes

The primary outcome was the prevalence of MDROs among patients undergoing ERCP in four countries. Prevalence was assessed per ERCP procedure, reflecting the infection-prevention perspective that each procedure represents a potential exposure of a duodenoscope to a patient colonised with an MDRO. The tested MDROs included extended-spectrum beta-lactamase-producing *Enterobacterales* (ESBL-E), carbapenemase-producing *Enterobacterales* (CPE), carbapenemase-producing *Pseudomonas aeruginosa* (CPPA), resistant *Acinetobacter calcoaceticus baumannii* complex (Acb-complex), vancomycin-resistant *Enterococcus faecium* (VRE), and methicillin-resistant *Staphylococcus aureus* (MRSA). The secondary outcome was to determine risk factors for MDRO-carriage in patients undergoing ERCP.

### Patient screening and eligibility criteria

Regardless of indication, adult patients aged 18 years or older undergoing ERCP, were eligible for participation. The ERCP could be performed in either the outpatient or inpatient setting. All patients provided written informed consent. Exclusion criteria included cases where the ERCP was not performed, a duodenoscope was not used, or the rectal swab (sample specifications below) was not collected. The procedure did not need to be completed successfully; early termination due to, for example, clinical instability or gastric food stasis did not lead to exclusion. For every excluded patient, a new patient was enrolled. Patients could participate twice, provided at least three months had passed since their previous inclusion.

Information on the numbers of participants who were approached and screened, declined participation, did not undergo ERCP, had incomplete sampling, or withdrew consent was not consistently retained across all participating sites. Consequently, it was not possible to produce a comprehensive study flow diagram of participant enrolment.

### Data collection

Baseline characteristics were extracted from medical records and entered into Castor electronic data capture (Castor, Amsterdam, the Netherlands). Data included demographics (age and sex), medical history, antibiotic or antacid/proton pump inhibitor (PPI) use within six months, clinical admissions exceeding 24 h within the past year, and endoscopic procedures performed in the past six months. Additionally, ERCP-specific details were documented.

### Sample collection

Before the ERCP, two dry eSwabs (Copan Italia SpA, Brescia, Italy) were used to collect rectal and throat-nose samples. The throat-nose sample was taken by rotating the swab for 5 s on each side of the throat near the tonsils, then inserting it 1–2 cm into each nostril and rotating against the nasal septum for 5 s. To minimise patient discomfort, the rectal swab was collected immediately before the ERCP, after the patient was sedated. The swab was inserted approximately four centimetres into the anus and rotated for 5 s, with discoloration indicating adequate sampling. Swabs were placed into transport tubes with 1 mL of Amies medium and sent to the microbiology lab. If immediate analysis was not possible, samples were refrigerated at 4 °C and processed within 48 h per manufacturer guidelines to preserve viability.

### Culturing protocols

All culturing protocols were standardised as much as possible across participating centres, though materials may have varied due to differences in product availability. Details on materials used at each site are provided in Supplementary appendix Table S1. Rectal and throat-nose swabs were cultured using organism-specific enrichment broths and selective agar plates. Identification and susceptibility testing were conducted using automated systems including MALDI-TOF (Bruker Daltonics, Bremen, Germany), VITEK 2 (bioMérieux, Marcy-l'Étoile, France), Phoenix (Becton, Dickinson and Company, Sparks, MD, USA), and MicroScan (Beckman Coulter, Brea, CA, USA), as available at each site. Results were interpreted using European Committee on Antimicrobial Susceptibility Testing (EUCAST) breakpoints.^16^ Confirmatory testing included ESBL group tests, the Carbapenem Inactivation Method (CIM), and PCR-based detection of resistance genes (e.g., *bla*_*OXA-48*_, *bla*_*KPC*_, *vanA/B*, *mecA/C*). Detailed protocols and site-specific testing variations are available in the Supplementary appendix.

### Sample size

The sample size was calculated using the method by Naing et al., designed to estimate population prevalence with high precision.^17^ An expected MDRO prevalence of 4.9% was assumed, based on preliminary data from a Dutch study later published with updated findings.^18^ A sample size of 286 patients was required to estimate this prevalence with 2.5% precision, defined as half the width of the 95% confidence interval. To account for variability in MDRO prevalence, the target was increased by 20%, setting a minimum of 343 patients per centre.

### Statistical analyses

Categorical variables were summarised as counts and percentages, while continuous variables were expressed as medians with interquartile ranges (Q1, Q3). Between-group differences were tested using the Chi-square test, or the Kruskal–Wallis test, as appropriate. Prevalence estimates were calculated per ERCP procedure (including repeat enrolments), with Wilson score 95% confidence intervals (CI). As a sensitivity analysis, prevalence estimates were also calculated excluding repeat enrolments, these results are presented in the Supplementary appendix. For risk-factor analysis, only the first participation per patient was included to avoid non-independence and over-representation, as most exposures are stable over three months while carriage status may vary. Twenty-five potential risk factors for preprocedural MDRO carriage were selected based on a literature review and group discussion. These included age, sex, comorbidities (chronic lung disease, diabetes mellitus, malignancies, congestive heart failure, end-stage renal disease), recent use of antibiotics (penicillins, including amoxicillin, amoxicillin with clavulanic acid, and piperacillin; cephalosporins; glycopeptides; carbapenems; and other classes), PPI or antacids, immunosuppressive medication, recent clinical admissions (hospital or long-term care), recent endoscopic procedures (ERCP, endoscopic ultrasound, gastroscopy, colonoscopy), and ERCP indication (chronic pancreatitis, pancreaticobiliary carcinoma, biliary stone disease, stenosis post-liver transplantation), adjusting for country of inclusion with the Netherlands as reference. Definitions of all variables are detailed in Table S2. Missing data were rare and limited to clinical admissions (3.2% for hospital, 0.3% for long-term care). A multivariate logistic regression model was fitted using the R package stats. Results were expressed as adjusted odds ratios (ORs) with 95% CIs. As a sensitivity analysis, we additionally fitted a modified Poisson regression model to estimate adjusted risk ratios (RRs), which are illustrated in the Supplementary appendix. A sensitivity analysis using multiple imputation (R mice package), confirmed no significant changes in the results (not shown). All analyses were performed using R (version 4.3.2), with p-values <0.05 considered statistically significant.

### Role of the funding source

The funder of the study had no role in study design, data collection, data analysis, data interpretation, or writing of the report. KvdP, MCV, JAS, BCGCS and MJB had full access to all the data in the study. These authors had final responsibility for the decision to submit the manuscript for publication.

## Results

### Patient inclusion

A total of 1154 patients, including 90 re-enrolled cases (1244 ERCP procedures), were analysed across four centres: 349 from India, 209 from Italy, and 343 each from the Netherlands and the US. In Italy, patient inclusion was halted after 209 cases due to logistical and personnel constraints.

### Patient characteristics

All patient characteristics were extracted from the electronic health records. Patient demographics and clinical practices varied widely across centres (Table 1). Significant inter-centre differences were identified in median age (p < 0.001), male prevalence (p = 0.001), and comorbidities including chronic lung disease, end-stage renal disease, malignancies, heart failure, and diabetes mellitus (all p < 0.001).

**Table 1:** Baseline characteristics of patients based on medical records review.

	Overall, N = 1244	India, N = 349	Italy, N = 209	Netherlands N = 343	US N = 343	p-values
**Patient characteristics**						
Re-enrolment	90 (7.3)	0	5 (2.4)	47 (13.7)	38 (11.1)	<0.001
Age (years), median [Q1, Q3]	60.00 [46.00, 71.00]	40.00 [30.00, 53.00]	70.00 [59.00, 79.00]	64.00 [53.00, 72.50]	64.00 [55.00, 73.00]	<0.001
Male	798 (64.1)	253 (72.5)	128 (61.2)	215 (62.7)	202 (58.9)	0.001
Female	446 (35.9)	96 (27.5)	81 (38.8)	128 (37.3)	141 (41.1)	0.001
**Medical history**						
Chronic lung disease (asthma, COPD)	194 (15.6)	1 (0.3)	18 (8.6)	43 (12.5)	132 (38.5)	<0.001
End-stage renal disease	27 (2.2)	0	15 (7.2)	6 (1.7)	6 (1.7)	<0.001
Malignancies	391 (31.4)	5 (1.4)	90 (43.1)	135 (39.4)	161 (46.9)	<0.001
Congestive heart failure	115 (9.2)	1 (0.3)	62 (29.7)	10 (2.9)	42 (12.2)	<0.001
Diabetes Mellitus	343 (27.6)	139 (39.8)	31 (14.8)	65 (19.0)	108 (31.5)	<0.001
**Immunosuppressive drug use (past six months)**	302 (24.3)	1 (0.3)	9 (4.3)	129 (37.6)	163 (47.5)	<0.001
Liver transplantation	110 (8.8)	1 (0.3)	0	70 (20.4)	39 (11.4)	<0.001
**Recent antibiotic use (past six months)**						
Penicillins	286 (23.0)	0	18 (8.6)	115 (33.5)	153 (44.6)	<0.001
Cephalosporins	227 (18.2)	6 (1.7)	10 (4.8)	85 (24.8)	126 (36.7)	<0.001
Glycopeptides	61 (4.9)	0	0	14 (4.1)	47 (13.7)	<0.001
Carbapenems	29 (2.3)	0	1 (0.5)	14 (4.1)	14 (4.1)	<0.001
Other antibiotics	255 (20.5)	12 (3.4)	6 (2.9)	108 (31.5)	129 (37.6)	<0.001
**PPI use (past six months)**	701 (56.4)	181 (51.9)	56 (26.8)	227 (66.2)	237 (69.1)	<0.001
**Antacids use (past six months)**	222 (17.8)	3 (0.9)	1 (0.5)	5 (1.5)	213 (62.1)	<0.001
**Clinical admission >24 h (past year)**	512 (41.2)	12 (3.4)	48 (23.0)	209 (60.9)	243 (70.8)	<0.001
Hospital (days), median [Q1, Q3]	7.00 [3.00, 17.00]	8.00 [4.25, 14.00]	6.00 [4.00, 8.50]	8.00 [3.00, 19.00]	7.00 [3.00, 16.00]	0.62
Long-term care facility (days), median [Q1, Q3]	15.00 [13.00, 38.00]	13.00 [13.00, 13.00]	11.00 [11.00, 11.00]	0.00 [0.00, 0.00]	16.00 [14.00, 43.00]	0.29
**Recent endoscopy (past six months)**						
ERCP	584 (46.9)	242 (69.3)	44 (21.1)	153 (44.6)	145 (42.3)	<0.001
Gastroscopy	73 (5.9)	0	11 (5.3)	34 (9.9)	28 (8.2)	<0.001
Colonoscopy	33 (2.7)	0	6 (2.9)	11 (3.2)	16 (4.7)	0.002
EUS	119 (9.6)	10 (2.9)	20 (9.6)	50 (14.6)	39 (11.4)	<0.001
**ERCP indication**						
(Suspected) biliary stone disease	312 (25.1)	76 (21.8)	72 (34.4)	74 (21.6)	90 (26.2)	0.003
Treatment of chronic pancreatitis	297 (23.9)	217 (62.2)	7 (3.3)	32 (9.3)	41 (12.0)	<0.001
(Suspected) pancreaticobiliary malignancy	292 (23.5)	23 (6.6)	84 (40.2)	88 (25.7)	97 (28.3)	<0.001
Obstructive jaundice of uncertain cause	189 (15.2)	11 (3.2)	37 (17.7)	81 (23.6)	59 (17.2)	<0.001
Treatment stenosis post-liver transplantation	68 (5.5)	1 (0.3)	0	35 (10.2)	32 (9.3)	<0.001
Bile leakage post-surgery	45 (3.6)	4 (1.1)	0	17 (5.0)	24 (7.0)	<0.001
(Suspected) ampullary malignancy	20 (1.6)	3 (0.9)	6 (2.9)	11 (3.2)	0	0.002
Other	21 (1.7)	14 (4.0)	3 (1.4)	5 (1.5)	0	

All values are numbers accompanied by percentages unless otherwise specified. p-values were calculated using the Chi-square test for categorical variables and the Kruskal–Wallis test for continuous variables.

COPD: chronic obstructive pulmonary disease, ERCP: endoscopic retrograde cholangiopancreatography, EUS: endoscopic ultrasound, PPI: proton pump inhibitor, US: United States.

Immunosuppressive drug use in the six months prior to ERCP varied significantly, highest in the US (163/343, 47.5%), as did prior liver transplantation rates, highest in the Netherlands (70/343, 20.4%; p < 0.001). The use of antibiotics and PPI differed significantly across centres (p < 0.001). The US reported the highest use of penicillins (153/343, 44.6%) and PPIs (237/343, 69.1%), while India reported no use of several antibiotics. Medical histories, including prior hospitalisations and endoscopic procedures, varied significantly (p < 0.001). Prior ERCP within six months was the most common procedure (584/1244, 46.9%).

The most common ERCP indications were (suspected) biliary stone disease (312/1244, 25.1%), chronic pancreatitis (297/1244, 23.9%), and (suspected) pancreaticobiliary malignancy (292/1244, 23.5%).

### Sample analysis

Significant variation in MDRO prevalence was observed across centres (Table 2). India reported the highest overall prevalence (83.1%, 290/349), followed by Italy (31.5%, 66/209), the US (20.1%, 69/343), and the Netherlands (10.8%, 37/343). Rectal screening identified ESBL-E in 337/1244 patients (27.1%), with significant differences between countries (p < 0.001). Prevalence was highest in India (70.2%, 245/349) and lowest in the US (4.4%, 15/343). CPE was identified in 94/1244 patients (7.6%), varying significantly by country (p < 0.001), with most cases in India (23.5%, 82/349) compared to the Netherlands (0.3%, 1/343) and the US (0.9%, 3/343). CPPA was rare (1/1244, 0.1%) and detected only in India. Resistant Acb-complex was found in 1/1244 patients (0.1%) in Italy. VRE was found in 83/1244 patients (6.7%), with significant inter-country differences (p < 0.001), with the highest prevalence in the US (10.8%, 37/343) and Italy (9.6%, 20/209), and no cases reported in the Netherlands. Throat-nose swabs were collected in 1244 cases. However, one sample from the Netherlands was lost during analysis. MRSA was detected in 39/1243 patients (3.1%), significantly different between countries (p < 0.001), with the highest prevalence in the US (7.9%, 27/343) and the lowest in the Netherlands (0.3%, 1/343). Among the 90 patients who were re-enrolled, four (4.4%) tested negative initially and positive on re-enrolment, while five (5.6%) tested positive initially and later tested negative. To assess the potential impact of re-enrolment, we performed a sensitivity analysis excluding second participations, which showed that overall prevalence estimates differed by less than 2% (Table S3). The MDRO species and detection frequencies are detailed in Table S4 (Supplementary appendix).

**Table 2:** Multidrug-resistant organisms detected through rectal and throat-nose screening.

	Overall, N = 1244	India, N = 349	Italy, N = 209	Netherlands N = 343	US N = 343	p-values
**Any MDRO**	462 (37.1, 34.5–39.9)	290 (83.1, 78.8–86.7)	66 (31.6, 25.7–38.2)	37 (10.8, 7.9–14.5)	69 (20.1, 16.2–24.7)	
**Rectal screening**						
ESBL-E	337 (27.1, 24.7–29.6)	245 (70.2, 65.2–74.8)	42 (20.1, 15.2–26.0)	35 (10.2, 7.4–13.9)	15 (4.4, 2.7–7.1)	<0.001
**Number of ESBL-E per patient (range)**	0–4	0–3	0–3	0–4	0–2	
CPE	94 (7.6, 6.2–9.2)	82 (23.5, 19.4–28.2)	8 (3.8, 1.9–7.4)	1 (0.3, 0–1.6)	3 (0.9, 0.3–2.5)	<0.001
**Number of CPE per patient (range)**	0–3	0–3	0–2	0–2	0–2	
CPPA	1 (0.1, 0–0.5)	1 (0.3, 0–1.6)	0	0	0	0.46
**Resistant Acb-complex**	1 (0.1, 0–0.5)	0	1 (0.5, 0–2.7)	0	0	0.18
VRE	83 (6.7, 5.4–8.2)	26 (7.4, 5.1–10.7)	20 (9.6, 6.3–14.3)	0	37 (10.8, 7.9–14.5)	<0.001
**Throat-nose screening**	1243	349	209	342	343	
MRSA	39 (3.1, 2.3–4.3)	5 (1.4, 0.6–3.3)	6 (2.9, 1.3–6.1)	1 (0.3, 0–1.6)	27 (7.9, 5.5–11.2)	<0.001

All values are numbers accompanied by percentages and 95% confidence intervals unless otherwise specified. The number of specific MDROs per patient is shown as a range only when patients carried more than one of a specific MDRO. The Chi-square test was used to calculate p-values.

Acb-complex: *Acinetobacter calcoaceticus baumannii complex,* ESBL-E: extended-spectrum beta–lactamase-producing *Enterobacterales,* CPE: carbapenemase-producing *Enterobacterales*, CPPA: carbapenemase-producing *Pseudomonas aeruginosa*, MDRO: multidrug resistant organisms, MRSA: methicillin-resistant *Staphylococcus aureus,* US: United States, VRE: vancomycin-resistant *Enterococcus faecium*.

### Risk factors for preprocedural MDRO carriage

**Fig. 1:**
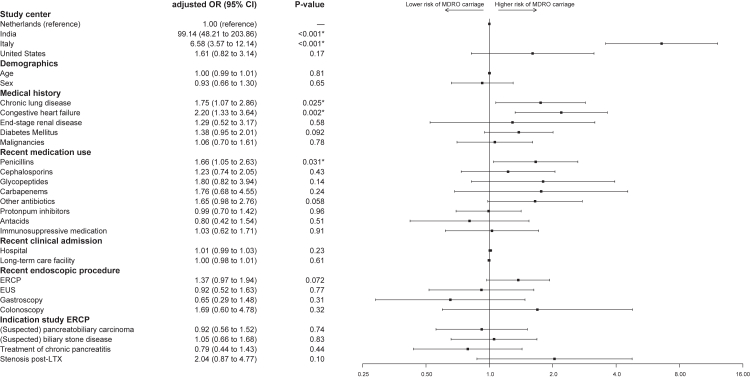
Forest plot illustrating the association between selected variables and MDRO carriage. Odds ratios (ORs) and 95% confidence intervals (CIs) were derived from multivariable logistic regression. The x-axis is displayed on a logarithmic ratio scale, centred at OR = 1 (no effect). Error bars indicate 95% CIs. Due to its outlier status the odds ratio of India is not displayed. ERCP: Endoscopic retrograde cholangiopancreatography, EUS: Endoscopic ultrasound, LTX: Liver transplantation, MDRO: Multidrug-resistant organisms, OR: Odds ratio. ∗ Statistically significant.

Geographic differences in MDRO carriage persisted after adjusting for patient characteristics, suggesting clinical factors alone do not explain the variation. Compared to the Netherlands, prevalence was significantly higher in India and Italy, but not in the US (Fig. 1). Among clinical factors, chronic lung disease (aOR 1.75, 95% CI:1.07–2.86, p = 0.025) and congestive heart failure (aOR 2.20, 95% CI:1.33–3.64, p = 0.002) were significantly associated with MDRO carriage. Recent use of penicillins (including amoxicillin, amoxicillin/clavulanic acid and piperacillin) was associated with increased odds of MDRO carriage (aOR 1.66, 95% CI:1.05–2.63, p = 0.031). Prior ERCP showed a non-significant trend (aOR 1.37, 95% CI:0.97–1.94, p = 0.072). The corresponding RRs from a sensitivity analysis using modified Poisson regression are presented in the Supplementary appendix (Figure S1). In this model, previous ERCP (aRR 1.15, 95% CI:1.01–1.31, p = 0.035) and the category ‘other antibiotics’ (aRR 1.37, 95% CI:1.04–1.81, p = 0.026) also reached statistical significance, while the overall pattern of associations remained similar.

## Discussion

This study assessed the prevalence of six MDROs among patients undergoing ERCP in four countries, highlighting risk of cross-contamination via duodenoscopes. Patients colonised with an MDRO represent a high-risk population for whom additional infection prevention measures, such as enhanced duodenoscope reprocessing, post-reprocessing microbiological surveillance, or the use of SUD, may be warranted. Overall, MDRO prevalence varied markedly, ranging from 83.1% (290/349) in India to 10.8% (37/343) in the Netherlands. In the US, gram-positive organisms such as VRE and MRSA predominated, whereas gram-negative bacteria, including ESBL-E and CPE, were more common in other centres. Among patient-related factors, chronic lung disease (aOR 1.75, 95% CI:1.07–2.86, p = 0.025), congestive heart failure (aOR 2.20, 95% CI:1.33–3.64, p = 0.002), and prior use of penicillins (aOR 1.66, 95% CI:1.05–2.63, p = 0.031) were significantly associated with preprocedural MDRO carriage. We acknowledge that, given the observed MDRO prevalence was considerably higher than assumed in the sample size calculation, odds ratios do not approximate relative risks and should not be interpreted as such, for adjusted RRs, see Supplementary Figure S1.

Studies examining MDRO prevalence in patients undergoing ERCP are limited. To date, only three prior US-based studies (Boston, Washington, Arizona), all from tertiary care centres and published between 2017 and 2018, have addressed this issue.^19^, ^20^, ^21^ These studies reported similarly low CPE prevalence compared to the results of our centre in the US (Pennsylvania, 3/343, 0.9%). However, Snyder et al. documented substantially lower VRE (3/189, 1.6%) and MRSA (1/189 0.5%) rates (37/343, 10.8% and 27/343, 7.9%, respectively).^19^ Notably, the use of enrichment broths in our study likely enhanced the sensitivity of MDRO detection.^22^ Moreover, as these studies were conducted 6–7 years ago, it is possible that MDRO prevalence has increased over time, reflecting broader trends in antimicrobial resistance.

A recent study, conducted at the same hospital in the Netherlands, reported an ESBL-E prevalence of 4.5% (43/1017) and an MRSA prevalence of 0.1% (1/1017) among patients who were hospitalised, with no cases of CPE, CPPA, resistant Acb-complex, or VRE.^18^ Our cohort of patients undergoing ERCP reflected these patterns, except for a higher ESBL-E rate at 9.5% (adjusted for re-enrolment, 28/296). This indicates patients undergoing ERCP in the Netherlands may be at greater risk for MDRO-carriage than the general hospitalised population. However, patients undergoing ERCP do not consistently show higher carriage rates than national averages. Although Italy reports some of the highest national antimicrobial resistance rates in Europe for CPE, VRE, and MRSA, our Italian centre showed lower CPE and MRSA prevalence, with only VRE rates comparable to national data.^23^ MDRO prevalence in our cohort of patients from India (Hyderabad) matched data from patients who were hospitalised in a general hospital in South India.^24^ Overall, patients undergoing ERCP are not uniformly at higher or lower risk of MDRO carriage compared to national prevalence data. Instead, the risk profile depends on local epidemiology, healthcare practices, and patient characteristics.

Our multivariate analysis identified chronic lung disease, congestive heart failure, and recent penicillin use as significantly associated with MDRO carriage. The associations of chronic lung disease and heart failure with MDRO carriage likely reflect a combination of frequent healthcare exposure and recurrent antibiotic use, for example during respiratory exacerbations or hospital admissions. Predictive models based on these factors may optimise preprocedural screening by targeting patients at greater risk of MDRO carriage. However, risk factors vary by specific MDRO types.^25^ Furthermore, considerable heterogeneity in patient populations and MDRO characteristics across centres complicates model development. Data reliability further limits predictive utility. For instance, the Indian centre treated many referred patients, which likely limited accurate medical or medication histories. Antibiotic use, typically sporadic and short-term, is likely underreported due to the absence of a national medication registry. In addition, the widespread availability of antibiotics without prescription, despite formal regulations, likely contributes to the high prevalence of MDRO in India and further complicates the reliable assessment of prior antibiotic use as a risk factor.^26^ Given these challenges, universally applicable predictive models, whether national or local, are unlikely to be feasible or effective for guiding infection control measures to prevent MDRO transmission via endoscopes.

The only validated method to assess duodenoscope contamination is microbiological culturing, and no point-of-care test is currently available or recommended by clinical practice guidelines.^27^^,^^28^ To prevent duodenoscope contamination following procedures on patients colonised with an MDRO, postprocedural strategies include enhanced surveillance (e.g., endoscope culturing) and intensified reprocessing, such as ethylene oxide sterilisation.^29^ Additionally, the use of SUD may be considered in patients colonised with an MDRO.^9^ A recent cost analysis estimated the break-even cost of implementing SUD for patients screened for an MDRO using Dutch and US healthcare system data.^9^ However, the MDRO prevalence used in that analysis was lower than in our study for both Dutch and US settings. Since higher MDRO prevalence increases the risk of MDRO-related DAI, the cost neutrality threshold for SUD may be slightly higher than previously estimated. Still, the actual risk of DAI remains a key uncertainty that limits accurate (cost-)effectiveness assessment of SUDs compared to other infection prevention strategies.^3^

Before implementing broad preprocedural MDRO screening, the ability of rectal and throat-nose swabs to estimate duodenoscope contamination risk should be critically evaluated. Duodenoscopes primarily contact the microbiome of the upper gastrointestinal tract, particularly the duodenum, which differs from the rectal and respiratory microbiota.^30^ The correlation between MDRO carriage at these screening sites and duodenal colonisation remains unknown. Therefore, future research should determine whether current screening methods reliably reflect duodenal MDRO presence.

This study has several limitations. First, it was conducted exclusively in tertiary care centres, which manage more complex and patient who are critically ill. This likely contributed to the high rates of immunosuppressive drug use and recent hospitalisations, limiting generalisability to local hospitals within the same countries. Second, baseline characteristics were derived from local medical records, which may vary in completeness. No supplemental data were collected from referring institutions, likely underestimating factors such as antibiotic use, particularly in India. Third, although six MDRO types were included, antibiotic resistance mechanisms and species continue to expand. Emerging pathogens like *Candida auris*, a growing concern in India, were not assessed.^31^ Therefore, overall MDRO prevalence may be underestimated. Fourth, despite efforts to standardise culturing protocols, complete uniformity was unachievable due to local resource constraints, potentially causing minor variation in detection sensitivity. Fifth, in India, ERCP volumes were markedly higher (up to 50/day), exceeding lab capacity and limiting daily enrolment. This may have introduced selection bias and limited the generalisability of findings from that centre. Moreover, the cohort of patients from India differed substantially from those at other sites in terms of demographics and ERCP indications, with patients who were younger and had fewer malignancies, which further limits comparability and generalisability across settings. Sixth, at the Italian site, 209 patients were enrolled versus the planned 343, yielding an MDRO prevalence of 31.6% (66/209, 95% CI 25.7–38.2%). This smaller sample size modestly widened the confidence interval (expected 26.8–36.6% with full enrolment). Seventh, data on participant screening, enrolment, and exclusions were not uniformly recorded across sites, preventing the creation of a complete study flow diagram. The absence of these data limits assessment of potential recruitment and sampling biases, thereby reducing certainty about the representativeness of the study population and the generalisability of the findings. Finally, substantial heterogeneity in patient characteristics and MDRO types across centres may have affected the identification and applicability of risk factors.

In conclusion, this international multicentre study demonstrates substantial variation in MDRO carriage rates and types among patients undergoing ERCP across countries. Our findings lay a vital groundwork for future research into the clinical benefits, cost-effectiveness, and environmental impact of preprocedural MDRO screening and targeted infection prevention strategies. Effective mitigation of MDRO transmission via endoscopes will require a balanced, context-specific approach that integrates rigorous reprocessing, microbiological surveillance, and comprehensive infection control, potentially including selective SUD use based on screening results.

## Contributors

KvdP: conceptualisation, data curation, formal analysis, investigation, methodology, project administration, validation, visualisation, writing – original draft, writing – review & editing. MCV: conceptualisation, funding acquisition, supervision, validation, writing – review & editing. HR: investigation, project administration, validation, writing – review & editing. JAS: methodology, supervision, validation, writing – review & editing. RAJP: formal analysis, methodology, writing – review & editing. DNR: supervision SYV: investigation, methodology, project administration, validation. MiS: investigation, supervision. SFM: investigation. AR: project administration, supervision. MaS: investigation. MC: investigation. MA: investigation. BAS: investigation, validation. RD: investigation. AS: project administration, supervision. BCGCS: methodology, supervision, validation, writing – review & editing. MJB: conceptualisation, funding acquisition, supervision, validation, writing – review & editing. KvdP, MCV, JAS, BCGCS and MJB had access to and verified the underlying study data.

## Data sharing statement

De-identified participant data underlying the findings reported in this study will be made available upon reasonable request following publication. Access will be granted only to qualified researchers for analyses consistent with the aims of the original study, after approval of a submitted research proposal and the signing of a data access agreement. Data sharing will be subject to any applicable institutional and regulatory requirements.

## Declaration of interests

KvdP received contributions toward PhD thesis printing costs from Copan Italia S.p.A., Pentax Medical, Wassenburg Medical, and Endoss. MCV received institutional research support from Pentax Medical. JAS served as chair of the IPC–MDROs working group (Dutch Collaborative Partnership for Infection Prevention Guidelines); an allowance was paid to Erasmus MC. AR acted as a consultant for Boston Scientific and Fujifilm and received honoraria for lectures/presentations from Fujifilm. MaS acted as a consultant for Boston Scientific and received honoraria for lectures/presentations from Boston Scientific and Olympus. MC received honoraria for lectures/presentations from Boston Scientific. MJB received research support from Boston Scientific, Cook Medical, Pentax Medical, and Interscope, and acted as a consultant/lecturer for Boston Scientific, Cook Medical, and Ecolab. HR, RAJP, BCGCMS, NR, SYV, MiS, SFM, MA, BAS, RD, and AS declare no competing interests.
