# Supplementary material for: Preprocedural screening for multidrug-resistant organisms in endoscopic retrograde cholangiopancreatography: an international, multicentre, cross-sectional observational study
Source: eClinicalMedicine. 2025 Nov 13;90:103627. doi: 10.1016/j.eclinm.2025.103627 (PMC12661358; doi:10.1016/j.eclinm.2025.103627)
Supplement: Supplementary Material [file mmc1.pdf]

Supplementary material

Preprocedural screening for multidrug-resistant organisms in endoscopic retrograde cholangiopancreatography: an international, multicentre, cross-sectional observational study

Koen van der Ploeg, MD[1,2], Margreet C. Vos, MD, PhD[2], Hardik Rughwani, MD[3], Juliëtte A. Severin, MD, PhD[2], Richard A. J. Post, PhD[4,5], D. Nageshwar Reddy, MD, DM, DSc[3], Sadhana Yelamanchili Veturi, MD[6], Mitnala Sasikala, PhD[7], Sana Fathima Memon[3], Alessandro Repici, MD[8,9], Marco Spadaccini, MD[8,9], Matteo Colombo, MD[8,9], Marta Andreozzi, MD[9], Bryan A. Stevens, MD[10], Rohit Das, MD[11], Adam Slivka, MD, PhD[11], Bibi C.G.C. Mason-Slingerland, MD, PhD[2]*, Marco J. Bruno, MD, PhD[1]*

Table of contents

Methods - Microbiological analysis

Supplementary Table S1: Overview of study materials used across participating centers for culturing protocols

Supplementary Table S2: Clinical and operational definitions of potential risk factors

Supplementary Table S3 Sensitivity analysis of multidrug-resistant organism prevalence in ERCP patients, excluding re-enrolled patients (patient-level prevalence)

Supplementary Table S4 Type and species of MDRO including their resistance genes detected through rectal and throat-nose screening

Supplementary Figure S1 Forest plot illustrating the association between selected variables and MDRO carriage. Risk ratios (RRs) and 95% confidence intervals (CIs) were derived from a multivariable modified Poisson regression model. The x-axis is displayed on a logarithmic ratio scale, centered at RR=1 (no effect). Error bars indicate 95% CIs. Due to its outlier status, the risk ratio for India is only partially displayed. ERCP: Endoscopic retrograde cholangiopancreatography, EUS: Endoscopic ultrasound, LTX: Liver transplantation, MDRO: Multidrug-resistant organisms, RR: Risk ratio. * Statistically significant

Microbiological analysis

Sample Preparation

The rectal swab was vortexed for ten seconds, and 150 µl of Amies medium was pipetted into three enrichment broths. To determine VRE, a broth with 8 mg/L of amoxicillin was used. For the detection of CPE, CPPA, and ESBL-E, a Tryptic Soy Broth (TSB) with 50 mg/L vancomycin was used. For resistant Acb-complex, TSB containing 2 mg/L ceftazidime and 50 mg/L vancomycin was employed. At the US site, ceftazidime was unavailable, and the CPE broth was also used to detect resistant Acb-complex. The throat-nose swab was similarly vortexed for 10 seconds, and 150 µL was added to TSB with 6.5% NaCl. All broths were incubated at 35 °C for 24 hours. After 24-hour incubation, 10 µL of each enrichment broth was inoculated onto the appropriate screening agar plates (Table S1). Plates were incubated for 48 hours at 35 °C in an O_2 atmosphere.

Identification

If growth was observed on selective screening agars, suspected strains were subcultured on Tryptic Soy Agar with 5% sheep blood and incubated overnight at 35 °C in a 5% CO_2 atmosphere. Bacterial identification at the study centers in the Netherlands, Italy, and the US was performed using a Matrix-Assisted Laser Desorption/Ionization Time-of-Flight (MALDI-TOF) analyzer (Bruker Daltonics, Bremen, Germany). In India, the VITEK 2 ID system (bioMérieux, Marcy-l'Étoile, France) was used. Susceptibility testing was conducted using the VITEK 2 system in the Netherlands and India, the Phoenix system (Becton, Dickinson and Company, Sparks, MD, USA) in Italy, and the MicroScan system (Beckman Coulter, Brea, CA, USA) in the US.

Confirmation

Antibiotic susceptibility breakpoints from the European Committee on Antimicrobial Susceptibility Testing (EUCAST) were used to identify suspected MDROs [16]. Suspected ESBL-E underwent group testing using ESBL confirmation kits to differentiate ESBL-E from AmpC-producing bacteria (Table S1). The kit used in India did not include cloxacillin and ceftazidime discs were unavailable in the US. In both cases cefepime discs were used instead. For suspected CPE or CPPA, carbapenemase activity was assessed using the Carbapenem Inactivation Method (CIM) test. Resistance genes were identified using GeneXpert (Cepheid, Maurens-Scopont, France) or an established lab-developed polymerase chain reaction (PCR). For carbapenemase-producing organisms, testing included the $bla_{OXA\text{-}48}$, bla_{KPC}, bla_{IMP}, bla_{VIM}, and bla_{NDM} genes. In suspected VRE cases, *vanA* and *vanB* genes were assessed via PCR. For MRSA, isolates from the screening agar were confirmed by cefoxitin resistance, followed by identification of *mecA* or *mecC* genes.

Table S1 Overview of study materials used across participating centers for culturing protocols

	Netherlands	India	Italy	United States
**Enrichment broths**				
Broth with vancomycin (50mg/l)	Self-prepared	Self-prepared	Self-prepared	Self-prepared
Broth with amoxicillin (8mg/l)	Self-prepared	Self-prepared	Self-prepared	Self-prepared
Broth with ceftazidime (2 mg/l) and vancomycin (50 mg/l)	Self-prepared	Self-prepared	Self-prepared	Not available
Trypticase Soy Broth + 6,5% NaCl	Tritium Microbiologie bv	Himedia	Becton Dickinson	Remel, Self-prepared
**ESBL-E screeningsplate**	Chromid ESBL, Biomerieux	HiCrome ESBL agar, Himedia	Brilliance ESBL agar, Oxoid Ltd	HardyCHROM ESBL agar, Hardy Diagnostics
**ESBL-E disk test confirmation kit**	Rosco, Taastrup, Denmark	Himedia	MASTDISCS Combi AmpC&ESBL ID, Mast Group	Rosco
**CPE and CPPA screeningsplate**	Chromid CarbaSmart, Biomerieux	Chomogenic KPC agar, Himedia	Brilliance CRE agar, Oxoid Ltd	HardyCHROM CRE agar, Hardy Diagnostics
**VRE screeningsplate**	Vanco Screening agar 6.0 mg. Oxoid Ltd	Chromogenic VRE plate Himedia	Brilliance VRE Agar, Oxoid Ltd	Remel Spectra VRE chromogenic medium
**Resistant *Acinetobacter calcoaceticus baumannii* complex screeningsplate**	Chromid ESBL, Biomerieux	HiCrome ESBL agar, Himedia	Brilliance ESBL agar, Oxoid Ltd	Included in CPE and CPPA protocol
**MRSA screeningsplate**	Chrom Agar MRSA II, Becton Dickinson	HiCrome MeReSa Agar, Himedia	MRSA2 Brilliance, Thermo Fisher	HardyCHROM MRSA, Hardy Diagnostics
**MacConkey agar plate**	Becton Dickinson	Himedia		Hardy Diagnostics
**Mueller Hinton agar plate**	Becton Dickinson	Himedia	Thermo Fisher Diagnostics	Hardy Diagnostics
**5% Sheep blood agar plate**	Becton Dickinson	Himedia	Becton Dickinson	Hardy Diagnostics
**Isolate identification**	MALDI-TOF, Bruker Daltonics	VITEK 2 ID system, bioMérieux	MALDI-TOF, Bruker Daltonics	MALDI-TOF, Bruker Daltonics
**Antibiotic susceptibility testing**	VITEK 2 ID system, Biomerieux	VITEK 2 ID system, Biomerieux	Phoenix system, Becton Dickinson	MicroScan system, Beckman Coulter

Headquarter locations of product companies: Beckman Coulter, Brea, California, U.S.; Becton Dickinson, Franklin Lakes, New Jersey, U.S.; bioMérieux, Marcy-l'Étoile, France; Bruker Daltonics, Bremen, Germany; Fisher Scientific, Hampton, New Hampshire, U.S.; Hardy Diagnostics, Santa Maria, California, U.S.; Himedia, Mumbai, India; Mast Group, Bootle, Merseyside, United Kingdom; Oxoid Ltd, Basingstoke, United Kingdom; Remel, Lenexa, Kansas, U.S.; Rosco, Taastrup, Denmark; Thermo Fisher Diagnostics, Waltham, Massachusetts, U.S.; Tritium Microbiologie bv, Eindhoven, Netherlands.

Table S2 Clinical and operational definitions of potential risk factors

Risk factor	Type	Units or levels	Definition
**Age**	Continuous	Years	Age of the patient at the time of study ERCP
**Sex**	Binary	Male/Female	Patient's sex
**Medical history**	Binary	Yes/No	Documented in medical records as a diagnosis, identified through medical notes, ICD codes, or medical correspondence (e.g., referral or discharge letters)
Chronic lung disease			Asthma, COPD or other chronic lung disease
Diabetes mellitus			Diabetes mellitus type I or II with active antidiabetic medication use
Malignancies			Any active or past malignancies for which patient received treatment either curative or palliative
Congestive heart failure			Any mention of past or active congestive heart failure
End-stage renal disease			GFR <15 mL/min/1.73 m², past or active dialysis or kidney transplantation
**Medication use**			Medication use for at least 24 hours within the past six months, as documented in medical records
Antibiotics			
Penicillin	Binary	Yes/No	e.g. penicillin, amoxicillin, ampicillin, piperacillin, amoxcillin-clavunate
Cephalosporins	Binary	Yes/No	1st, 2nd, 3rd or 4th generation
Glycopeptides	Binary	Yes/No	e.g. vancomycin, teicoplanin
Carbapenems	Binary	Yes/No	e.g. carbapenem, meropenem, imipenem
Other	Binary	Yes/No	e.g. tetracyclins, macrolides, co-trimoxazole, quinolones
Proton pump inhibitors	Binary	Yes/No	e.g. esomeprazole, rabeprazole, pantoprazole, omeprazole, lansoprazole, other
Antacids	Binary	Yes/No	e.g. magnesium based, sodium based, calcium based, aluminum based
Immunosuppressive medication	Binary	Yes/No	e.g. corticosteroids, janus kinase inhibitors, calcineurin inhibitors, mTOR inhibitors, IMDH inhibitors, biologics, monoclonal antibodies
**Clinical admission**			Documented admission of at least 24 hours in medical records or correspondence
Hospital	Continuous	Days	
Long term care facility	Continuous	Days	
**Endoscopic procedures**			Endoscopic procedures within the past six months documented in medical records, ICD codes, or correspondence
ERCP	Binary	Yes/No	
Endoscopic ultrasound	Binary	Yes/No	
Gastroscopy	Binary	Yes/No	
Colonoscopy	Binary	Yes/No	
**ERCP indication**			Indication of study ERCP, as documented in the order or ERCP report
Treatment of chronic pancreatitis	Binary	Yes/No	
(Suspected) pancreaticobiliary malignancy	Binary	Yes/No	
(Suspected) biliary stone disease	Binary	Yes/No	
Treatment of stenosis post-liver transplantation	Binary	Yes/No	

COPD: chronic obstructive pulmonary disease, ERCP: Endoscopic retrograde cholangiopancreatography, GFR: glomerular filtration rate, ICD: international classification of diseases, IMDH: inosine monophosphate dehydrogenase

Table S3 Sensitivity analysis of multidrug-resistant organism prevalence in ERCP patients, excluding re-enrolled patients (patient-level prevalence)

	Overall, N= 1154	India, N= 349	Italy, N=204	Netherlands N=296	US N=305	p-values
**Any MDRO**	448 (38.9, 36.1-41.7)	290 (83.1, 78.8-86.7)	66 (32.4, 26.3-39.0)	30 (10.1, 7.2-14.1)	62 (20.3, 16.2-25.2)	
**Rectal screening**						
ESBL-E	328 (28.4, 25.9-31.1)	245 (70.2, 65.2-74.8)	42 (20.6, 15.6-26.7)	28 (9.5, 6.6-13.3)	13 (4.3, 2.5-7.2)	<0.001
CPE	94 (8.1, 6.7-9.9)	82 (23.5, 19.4-28.2)	8 (3.9, 2.0-7.5)	1 (0.3, 0-1.9)	3 (1.0, 0.3-2.9)	<0.001
CPPA	1 (0.1, 0-0.5)	1 (0.3, 0-0.2)	0	0	0	0.51
Resistant Acb-complex	1 (0.1, 0-0.5)	0	1 (0.5, 0-2.7)	0	0	0.20
VRE	80 (6.9, 5.6-8.5)	26 (7.4, 5.1-10.7)	20 (9.8, 6.4-14.7)	0	34 (11.1, 8.1-15.2)	<0.001
**Throat-nose screening**						
MRSA	35 (3.0, 2.2-4.2)	5 (1.4, 0.6-3.3)	6 (2.9, 1.4-6.3)	1 (0.3, 0-1.9)	23 (7.5, 5.1-11.1)	<0.001

Acb-complex: *Acinetobacter calcoaceticus baumannii complex,* ESBL-E: extended-spectrum beta–lactamase-producing *Enterobacterales,* CPE: carbapenemase-producing *Enterobacterales*, CPPA: carbapenemase-producing *Pseudomonas aeruginosa*, MDRO: multidrug resistant organisms, MRSA: methicillin-resistant *Staphylococcus aureus,* US: United States, VRE: vancomycin-resistant *Enterococcus faecium*. All values are numbers accompanied by percentages.

Table S4 Type and species of MDRO including their resistance genes detected through rectal and throat-nose screening

	Overall	India	Italy	Netherlands	United States
**Rectal screening**					
**Total number rectal MDRO**	658 (100)	480 (100)	77 (100)	44 (100)	57 (100)
**ESBL-E**	451 (68.5)	346 (72.1)	47 (61.0)	42 (95.5)	16 (28.1)
Escherichia coli	284 (43.2)	216 (45.0)	36 (46.8)	25 (56.8)	7 (12.3)
Klebsiella pneumoniae	123 (18.7)	108 (22.5)	4 (5.2)	7 (15.9)	4 (7.0)
Enterobacter cloacae complex	12 (1.8)	9 (1.9)	1 (1.3)	1 (2.3)	1 (1.8)
Citrobacter freundii complex	8 (1.2)	1 (0.2)	0	3 (6.8)	4 (7.0)
Morganella morganii	6 (0.9)	5 (1.0)	1 (1.3)	0	0
Proteus mirabilis	3 (0.5)	1 (0.2)	2 (2.6)	0	0
Klebsiella aerogenes	3 (0.5)	2 (0.4)	1 (1.3)	0	0
Proteus vulgaris	2 (0.3)	0	1 (1.3)	1 (2.3)	0
Serratia marcescens	2 (0.3)	1 (0.2)	0	1 (2.3)	0
Citrobacter amalonaticus complex	1 (0.2)	0	0	1 (2.3)	0
Citrobacter braakii	1 (0.2)	0	1 (1.3)	0	0
Citrobacter koseri	1 (0.2)	1 (0.2)	0	0	0
Citrobacter sedlakii	1 (0.2)	0	0	1 (2.3)	0
Hafnia alvei	1 (0.2)	0	0	1 (2.3)	0
Proteus hauseri	1 (0.2)	0	0	1 (2.3)	0
Serratia fonticola	1 (0.2)	1 (0.2)	0	0	0
Serratia odorifera	1 (0.2)	1 (0.2)	0	0	0
**CPE**	122 (18.5)	107 (22.3)	9 (11.7)	2 (4.5)	4 (7.0)
Escherichia coli	64 (9.7)	60 (12.5)	2 (2.6)	2 (4.5)	0
Klebsiella pneumoniae	49 (7.4)	42 (8.8)	6 (7.8)	0	1 (1.8)
Citrobacter freundii complex	2 (0.3)	0	0	0	2 (3.5)
Citrobacter koseri	1 (0.2)	1 (0.2)	0	0	0
Citrobacter sedlakki	1 (0.2)	1 (0.2)	0	0	0
Klebsiella aerogenes	1 (0.2)	0	1 (1.3)	0	0
Klebsiella oxytoca	1 (0.2)	1 (0.2)	0	0	0
Raoultella ornithinolytica	1 (0.2)	0	0	0	1 (1.8)
Salmonella enterica	1 (0.2)	1 (0.2)	0	0	0
Serratia marcescens	1 (0.2)	1 (0.2)	0	0	0
CPE resistance genes					
KPC,NDM	41 (6.2)	41 (8.5)	0	0	0
NDM	37 (5.6)	34 (7.1)	1 (1.3)	2 (4.5)	0
KPC,NDM,VIM	11 (1.7)	11 (2.3)	0	0	0
KPC	9 (1.4)	0	6 (7.8)	0	3 (5.3)
KPC,NDM,IMP	6 (0.9)	6 (1.3)	0	0	0
KPC,NDM,OXA-48	3 (0.5)	3 (0.6)	0	0	0
NDM,VIM	3 (0.5)	3 (0.6)	0	0	0
NDM,IMP	2 (0.3)	2 (0.4)	0	0	0
NDM,KPC,OXA-48	2 (0.3)	2 (0.4)	0	0	0
KPC,NDM,IMP,OXA-48	1 (0.2)	1 (0.2)	0	0	0
NDM,OXA-48	1 (0.2)	1 (0.2)	0	0	0
OXA 48	1 (0.2)	0	0	0	1 (1.8)

VIM,IMP,NDM,KPC	1 (0.2)	1 (0.2)	0	0	0
VIM,NDM,OXA-48	1 (0.2)	1 (0.2)	0	0	0
Unknown	1 (0.2)	0	1 (1.3)	0	0
**CPPA (*Pseudomonas aeruginosa*)**	1 (0.2)	1 (0.2)	0	0	0
CPPA resistance genes					
KPC, NDM, IMP	1 (0.2)	1 (0.2)	0	0	0
**Resistant Acb-complex**	1 (0.2)	0	1 (1.3)	0	0
**VRE (*Enterococcus faecium*)**	83 (12.6)	26 (5.4)	20 (26.0)	0	37 (64.9)
VRE resistance genes					
VanA	80 (12.2)	26 (5.4)	17 (22.1)	0	37 (64.9)
VanA + VanB	2 (0.3)	0	2 (2.6)	0	0
VanB	1 (0.2)	0	1 (1.3)	0	0
**Throat-nose screening**					
**MRSA (*Staphylococcus aureus*)**	39 (100)	5 (100)	6 (100)	1 (100)	27 (100)
MRSA resistance genes					
mecA	38 (97.4)	4 (80.0)	6 (100)	1 (100)	27 (100)
mecA, mecC	1 (2.6)	1 (20.0)	0	0	0

Acb-complex: *Acinetobacter calcoaceticus baumannii complex,* ESBL-E: extended-spectrum beta–lactamase-producing *Enterobacterales,* CPE: carbapenemase-producing *Enterobacterales*, CPPA: carbapenemase-producing *Pseudomonas aeruginosa*, MDRO: multidrug resistant organisms, MRSA: methicillin-resistant *Staphylococcus aureus,* VRE: vancomycin-resistant *Enterococcus faecium*. All values are numbers accompanied by percentages.

Forest plot illustrating the association between selected variables and MDRO carriage. Risk ratios (RRs) and 95% confidence intervals (CIs) were derived from a multivariable modified Poisson regression model. The x-axis is displayed on a logarithmic ratio scale, centered at RR=1 (no effect). Error bars indicate 95% CIs. Due to its outlier status, the risk ratio for India is only partially displayed.

	adjusted RR (95% CI)	P-value
**Study center**		
Netherlands (reference)	1.00 (reference)	—
India	13.05 (8.61 to 19.76)	<0.001*
Italy	4.10 (2.60 to 6.45)	<0.001*
United States	1.43 (0.88 to 2.34)	0.15
**Demographics**		
Age	1.00 (1.00 to 1.00)	0.86
Sex	0.99 (0.88 to 1.13)	0.90
**Medical history**		
Chronic lung disease	1.43 (1.03 to 1.98)	0.031*
Congestive heart failure	1.69 (1.25 to 2.28)	0.001*
End-stage renal disease	1.20 (0.73 to 1.95)	0.47
Diabetes Mellitus	1.11 (0.98 to 1.26)	0.12
Malignancies	1.06 (0.80 to 1.39)	0.70
**Recent medication use**		
Penicillins	1.49 (1.08 to 2.05)	0.015*
Cephalosporins	1.20 (0.89 to 1.61)	0.22
Glycopeptides	1.41 (0.86 to 2.29)	0.17
Carbapenems	1.45 (0.85 to 2.48)	0.17
Other antibiotics	1.37 (1.04 to 1.81)	0.026*
Protonpum inhibitors	1.00 (0.89 to 1.13)	0.97
Antacids	0.92 (0.60 to 1.39)	0.68
Immunosuppressive medication	1.06 (0.74 to 1.53)	0.73
**Recent clinical admission**		
Hospital	1.01 (1.00 to 1.01)	0.27
Long-term care facility	1.00 (0.99 to 1.00)	0.55
**Recent endoscopic procedure**		
ERCP	1.15 (1.01 to 1.31)	0.035*
EUS	0.94 (0.66 to 1.33)	0.71
Gastroscopy	0.73 (0.42 to 1.26)	0.26
Colonoscopy	1.43 (0.76 to 2.67)	0.27
**Indication study ERCP**		
(Suspected) pancreatobiliary carcinoma	0.97 (0.75 to 1.26)	0.83
(Suspected) biliary stone disease	1.04 (0.84 to 1.29)	0.74
Treatment of chronic pancreatitis	0.98 (0.82 to 1.18)	0.85
Stenosis post-LTX	1.60 (0.98 to 2.61)	0.061

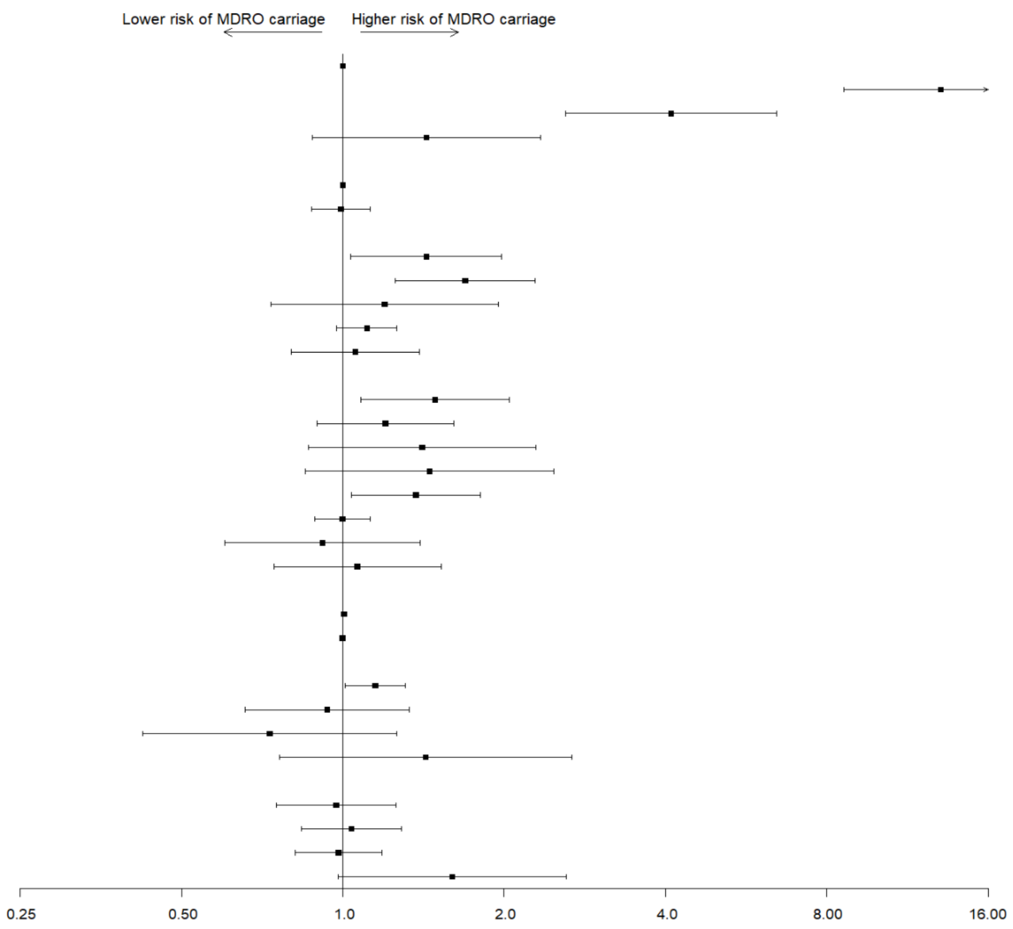

ERCP: Endoscopic retrograde cholangiopancreatography, EUS: Endoscopic ultrasound, LTX: Liver transplantation, MDRO: Multidrug-resistant organisms, RR: Risk ratio. * Statistically significant
